# Social Justice and the Promotion of the Common Good in Medical Missions to Low-Resourced Countries

**DOI:** 10.5334/aogh.2519

**Published:** 2019-06-17

**Authors:** Andrea Vicini

**Affiliations:** 1 Boston College, US

## Abstract

The article highlights the importance of critically examining medical missions to low-resourced countries in light of a bioethical focus on social justice and the promotion of the common good.

Health is an indispensable but scarce good, and health disparities characterize our world today. Inequalities within populations and countries depend on human diversity and social arrangements. These inequalities become inequities when, because of diversity, whether physical, racial, or class, people suffer discrimination, marginalization, and stigma, and they lack availability and access to what is essential for individual and social thriving.^1^ In the case of health, society tolerates inequalities, but people of goodwill should not accept inequities because they threaten individual and collective health, well-being, and flourishing. Hence, to address inequitable health differences, healthcare practitioners strive to promote greater health equity.

This vision guides healthcare practice and its commitment to care for people in need. However, how do we promote health globally and locally by addressing the health needs of countries, populations, and communities with limited health resources and scarce health infrastructures? In the Global North, we feel the responsibility of helping those who are less advantaged in the Global South, by sharing with them our healthcare expertise (e.g., surgical procedures performed *in loco*) and part of our resources (from drugs to diagnostic and therapeutic equipment). The health needs of so many people are so great and urgent that no matter what we will do and provide will be beneficial—even if it will require bending a few laws or avoiding following legal and healthcare standard procedures both in our own countries and in the receiving countries. At least, this is how things go in many healthcare missions in low-resourced countries.

Virginia Rowthorn et al. rightly tell us that this reasoning, despite being animated by the desire to help, is both flawed and dangerous. A double standard cannot inform our healthcare practice. While in the Global North we rightly uphold demanding and necessary ethical standards and carefully follow legal requirements and procedures, we cannot be careless in violating them in the Global South, in the name of the local lack of healthcare resources, infrastructures, and delivery systems.

In 1997, Marcia Angell, at that time executive editor of the *New England Journal of Medicine*, sounded her ethical alarm on the ethical double standards in clinical trials. As in the case of the infamous Tuskegee study of untreated syphilis, which spanned from 1932 to 1972, “the ethics of ongoing trials in the Third World of regimens to prevent the vertical transmission of human immunodeficiency virus (HIV) infection” raised her concerns.^2^ Studying zidovudine, “All except one of the trials employ placebo-treated control groups, despite the fact that zidovudine has already been clearly shown to cut the rate of vertical transmission greatly and is now recommended in the United States for all HIV-infected pregnant women.”^3^ The rationale to choose placebo-treated control groups was disturbing: “Women in the Third World would not receive antiretroviral treatment anyway, so the investigators are simply observing what would happen to the subjects’ infants if there were no study.”^4^ Hence, for Angell, it was necessary a “commitment to the highest ethical standards, no matter where the research is conducted, and sponsoring agencies need to enforce those standards, not undercut them.”^5^ Hence, “Human subjects in any part of the world should be protected by an irreducible set of ethical standards, including the requirements that they not be subjected to unreasonable risks and that they be asked for informed consent to participate.”^6^

In light of their interdisciplinary expertise, Rowthorn et al. tell us that today multiple ethical and legal violations occur not only in clinical trials but also in short-term global health experiences. The willingness to help people in disadvantaged locations that lack sufficient health services is praiseworthy, but it does not justify any disregard nor violation of already established ethical standards and requirements in medical practice. To provide care abroad, where healthcare is a scarce commodity, does not justify any involvement of students who lack appropriate training and certifications, and demands great care in examining how drugs are exported and distributed.

In their remarkable and well-documented legal and ethical analysis of short-term global health experiences, Rowthorn et al. articulated what, in ethical terms, is a vision of social justice in healthcare practice that is centered on a preferential option for those who are disadvantaged, discriminated, and marginalized, and that aims at promoting their health—their common good—in ways that are ethically and legally sound.

The common good allows the *ultimate realization of individual and social capabilities*. It aims at *individual and collective flourishing* by encompassing all social goods (i.e., spiritual, moral, relational, and material), for all human beings.^7^ In a world scarred by social, economic and political inequities, both in the Global North^8^ and in the Global South,^9^ *social justice* pursues the common good. Because it depends on human dignity, the common good aims at achieving a social coexistence characterized by authentic *solidarity*, which implies the readiness to care for those who, in civil society, have greater needs and are less advantaged.

Concretely, first, medical missions abroad should be part of the ongoing *local dynamisms* aimed at implementing and strengthening local healthcare resources. Second, the *agenda of health priorities* that the health mission will fulfill should be set by the local communities, with their leaders, healthcare practitioners, and institutions. To avoid paternalistic, colonial, and imperialistic approaches, and to foster a collaborative interaction based on equality and mutuality, the local empowerment is indispensable. Third, the health missions should fit harmoniously within a more *comprehensive promotion of health*. On the one hand, follow-up and continuity should be assured. On the other hand, health is not an isolated good. It depends on many social determinants that positively strengthen health in specific contexts (e.g., education, jobs, social infrastructures—from sanitation to roads—food quality and availability, environmental quality). Fourth, *critical evaluation and assessment* of each health mission should lead to further planning. The goal is to accompany the local communities, neither replacing them, not even for the very short length of medical missions, nor abandoning them to their own fate after the health mission ended.

“Not above the law” and for the common good: the health of many people across the planet demands such an inclusive and ambitious commitment.

## Footnotes

See Kristen Smith, “The Problematization of Medical Tourism: A Critique of Neoliberalism,” *Developing World Bioethics* 12, no. 1 (2012) 1–8.

Marcia Angell, “The Ethics of Clinical Research in the Third World,” *New England Journal of Medicine* 337, no. 12 (1997) 847–49, at 847.

Ibid.

Ibid.

Ibid. 849.

Marcia Angell, “Ethical Imperialism? Ethics in International Collaborative Clinical Research,” *New England Journal of Medicine* 319, no. 16 (1988) 1081–83, at 1083.

The common good is “the range of economic, political and social conditions necessary for the flourishing of individual members of a community, for respect of fundamental rights, and/or for achieving appropriate common goals.” Catholic Bishops of England and Wales Scotland and Ireland Joint Committee on Bio-Ethical Issues, “Catholic Social Teaching and the Allocation of Healthcare,” in *Healthcare Allocation: An Ethical Framework for Public Policy*, eds. Anthony Fisher and Luke Gormally (London: Linacre Centre, 2001) 145–61, at 154. See also Vatican Council II, Pastoral Constitution on the Church in the Modern World *Gaudium et Spes*, (1965), http://www.vatican.va/archive/hist_councils/ii_vatican_council/documents/vat-ii_cons_19651207_gaudium-et-spes_en.html no. 26.

See Kate Ward and Kenneth R. Himes, O.F.M., “‘Growing Apart’: The Rise of Inequality,” *Theological Studies* 75, no. 1 (2014) 118–32.

See Agbonkhianmeghe E. Orobator, S.J., “*Caritas in Veritate* and Africa’s Burden of (under)Development,” ibid. 71, no. 2 (2010) 320–34.

